# Effects of low birth weight, maternal smoking in pregnancy and social class on the phenotypic manifestation of Attention Deficit Hyperactivity Disorder and associated antisocial behaviour: investigation in a clinical sample

**DOI:** 10.1186/1471-244X-7-26

**Published:** 2007-06-20

**Authors:** Kate Langley, Peter A Holmans, Marianne BM van den Bree, Anita Thapar

**Affiliations:** 1 Department of Psychological Medicine, School of Medicine, Cardiff University, Heath Park, Cardiff, CF14 4XN, UK

## Abstract

**Background:**

Attention Deficit Hyperactivity Disorder (ADHD) is a genetically influenced condition although indicators of environmental risk including maternal smoking during pregnancy, low birth weight and low social class have also been found to be associated with the disorder.

ADHD is a phenotypically heterogeneous disorder in terms of the predominant symptom types (inattention, hyperactive-impulsivity), their severity and comorbidity, notably Conduct Disorder. It is possible that these different clinical manifestations of the disorder may arise because of the differing effects of the environmental indicators of environmental risk. We set out to test this hypothesis.

**Methods:**

In a sample of 356 children diagnosed with ADHD, we sought to investigate possible effects of three indicators of environmental risk – maternal smoking during pregnancy, birth weight and social class – on comorbid Conduct Disorder, conduct disorder symptoms and inattentive and hyperactive-impulsive symptom severity.

**Results:**

Multiple regression analysis revealed that, after controlling for significant covariates, greater hyperactive-impulsive symptom severity was significantly associated with maternal smoking during pregnancy (r^2 ^= 0.02, Beta = 0.11, t = 1.96, p = 0.05) and social class (r^2 ^= 0.02, Beta = 0.12, t = 2.19, p = 0.03) whilst none of the environmental risk indicators significantly predicted number of inattentive symptoms. Conduct Disorder symptoms were positively predicted by maternal smoking in pregnancy (r^2 ^= 0.04, Beta = 0.18, t = 3.34, p = 0.001) whilst both maternal smoking during pregnancy and social class significantly predicted a diagnosis of Conduct Disorder (OR = 3.14, 95% CI: 1.54, 6.41, Wald = 9.95, p = 0.002) and (OR = 1.95 95% CI: 1.18, 3.23 Wald = 6.78, p = 0.009) respectively.

**Conclusion:**

These findings suggest that indicators of environmental risk, in this instance maternal smoking in pregnancy and environmental adversity indexed by lower social class, independently influence the clinical presentation of the ADHD phenotype. Other types of study design are needed to investigate whether these associations between indicators of environmental risk factors and ADHD clinical heterogeneity are attributable to causal risk effects and to further establish the magnitude of these effects. These findings have implications, not only for our understanding of the aetiology of ADHD, but may also be of clinical value, enabling the identification of individuals who are at higher risk of problematic behaviours in ADHD, notably conduct disorder, to enable earlier, targeted risk reduction strategies.

## Background

Attention Deficit Hyperactivity Disorder (ADHD) is a neurodevelopmental disorder which affects between three and five percent of school aged children [1]. Characterised by excessive levels of hyperactivity, inattention and impulsivity which lead to impaired home, peer and school functioning, ADHD is one of the most common reasons for referral to Child and Adolescent Mental Health Services [2]. The negative impact of ADHD on peer and family relations, as well as poor academic performance [3] means that ADHD has a detrimental effect on individuals, their families and society as a whole.

Although there is a large genetic contribution to the aetiology of ADHD, twin studies indicate that environmental factors are also important. Shared and unique environmental factors account for between 12 and 40% of the variance in twin ADHD scores [4]. Environmental factors may also exert important risk effects through interplay with genetic risk factors [5] Pre, peri- and postnatal factors as well as exposure to psychosocial adversity throughout childhood have been found to be associated with symptoms and a diagnosis of ADHD,[6] although establishing whether these indicators of environmental risk have causal risk effects on ADHD remains a challenge.

Low birth weight and maternal smoking in pregnancy are the indicators of environmental risk that have been found to be most robustly associated with ADHD with results of pooled or meta-analyses showing significant evidence of association [7,8].

Numerous studies have reported increased rates of ADHD diagnosis and symptoms in case control studies of low or very low birth weight children [9-11]. McCormick and colleagues (1996) [12] have also demonstrated that hyperactivity symptom scores increase as birth weight decreases, regardless of whether birth weight is considered as a categorical or a continuous measure. These findings are further supported by a meta-analysis of 16 case-control studies of low birth weight and ADHD [7]. Bhutta and colleagues found a significant combined calculated risk ratio of 2.64 (95% CI: 1.85, 3.78 p < 0.001), indicating that low birth weight represents a significant indicator of risk for ADHD.

Over the past 20 years, population based epidemiological studies [13-15] and case-control studies [16,17] have indicated higher rates of ADHD symptoms and diagnosis in offspring of mothers who smoked during pregnancy. The relationship appears to be dose-dependent; the more cigarettes a mother smokes during pregnancy, the greater the risk to her child. A recent pooled analysis of case-control studies found a significant association between maternal smoking during pregnancy and a diagnosis of ADHD, with an Odds Ratio of 2.39 (95% CI: 1.61, 3.52 p < 0.0001) [8].

Beyond early (ante- and post-natal) development, ADHD has also been shown to be associated with poverty, lower family income and social class. Epidemiological studies in the UK, USA and across the world [18-20] have demonstrated that symptoms of ADHD are more common in children living in poverty or from lower social class families. Similarly, clinical studies have shown that ADHD children are more likely to be from low social class families than healthy controls [21,22].

The relations between indicators of environmental risk and ADHD are complicated. These indicators of environmental risk tend to co-occur and to be related to different behavioural outcomes that have also been associated with ADHD. Mothers who smoke are more likely to be of a lower social class and to give birth to lighter babies. [23] Similarly, maternal smoking during pregnancy, low birth weight and social class have all been demonstrated to predict factors associated with ADHD including Conduct Disorder symptoms, [15] verbal IQ [7] and gender. [24] However, the associations between these indicators of environmental risk and ADHD have been demonstrated to remain significant in studies which control for other covariates [13,16,19].

ADHD is a clinically heterogeneous disorder and there is evidence to suggest that this phenotypic heterogeneity may index underlying aetiological heterogeneity. That is, it may be that different risk pathways lead to different patterns of symptoms, symptom severity, or other clinical manifestations of the disorder. One of the main observed forms of heterogeneity lies in the pattern of comorbidity. ADHD is frequently found to co-occur with a number of other disorders, most commonly Oppositional Defiant Disorder (ODD) and Conduct Disorder (CD). Between 43 and 93% of children with ADHD are estimated to have one of these disorders. [25] There is a wealth of evidence to suggest that individuals with comorbid antisocial behaviour, in addition to ADHD, fare much worse than those with just one disorder, on a wide range of measures. In comparison to those with ADHD alone, individuals with a comorbid CD have a greater number of ADHD symptoms [26,27], more social impairments [26] and are at greater risk of other psychiatric disorders and substance abuse in later life [28,29].

Clinical heterogeneity also arises because of the preponderance of different types of ADHD symptoms, that is; Hyperactive-Impulsive symptoms and Inattentive symptoms [30] and their severity. There is support for the distinction of Hyperactive-Impulsive and Inattentive symptom groups in ADHD because they lead to slightly different types of social impairments [31] and behaviour styles. [31] For example, hyperactive-impulsive symptoms are more likely to lead to comorbid antisocial behaviour problems than inattentive symptoms. [31] Currently, DSM-IV criteria for ADHD allows subtypes of the disorder where only symptoms from one of the dimensions are present (ADHD predominantly Inattentive type and ADHD predominantly hyperactive-impulsive type) as well as the combined type characterised by symptoms from both dimensions [30].

Family and twin studies suggest that the phenotypic heterogeneity characterised by ADHD symptom subtypes and comorbid conduct disorder or conduct disorder symptoms indexes underlying aetiological heterogeneity [32]. There is increasing evidence to suggest that genetic risk factors contribute to this aetiological heterogeneity. For example, specific gene variants have been found to influence conduct disorder symptoms in ADHD [4] as well as symptom subtype severity [33]. However the question of whether or not indicators of environmental risk have an effect on the clinical manifestation of ADHD has not yet been considered, to our knowledge. We set out to investigate whether indicators of environmental risk previously found to be associated with ADHD, specifically birth weight, maternal smoking in pregnancy and social class (as an index of social adversity) are associated with conduct disorder, conduct disorder symptoms, hyperactive-impulsive and inattention symptom severity in a clinical sample of 356 children with ADHD. This question is important for our understanding of the aetiology of ADHD, but also for clinical practice, because it will contribute to our ability to identify individuals at higher risk of problematic behaviours in ADHD, thus enabling earlier, targeted risk reduction strategies. As hypothesised, we found that indicators of environmental risk appeared to have a significant effect on the ADHD phenotype.

## Methods

356 British Caucasian children aged between 6 and 16 years with a diagnosis of ADHD (subsequently confirmed by research diagnostic interview assessments) were referred to the study by Child and Adolescent Psychiatrists and Paediatricians in the Greater Manchester, South Wales and Avon areas of the UK. Following informed written consent from parents and assent from children, individuals underwent a full assessment procedure. Parents (typically the mother) were interviewed regarding their child's ADHD, ODD and CD symptoms using the Child and Adolescent Psychiatric Assessment (CAPA). [34] The CAPA is a semi-structured interview based on DSM-IV criteria, with good reliability. In this study, reliability between trained, supervised interviewers was excellent, with Kappa values of 1.00 for ADHD diagnosis. CAPA ratings also provided total hyperactive-impulsive, inattentive, ODD and CD symptom score counts. Information on the presence of ADHD symptoms or impairment within a school setting was obtained using the Child ADHD Teacher Telephone Interview (ChATTI) [35]. Individuals were excluded from the study if they did not live with at least one biological parent, had a Full Scale IQ of less than 70 (assessed using the WISC-III), [36] had Tourette's Syndrome, a Pervasive Developmental Disorder or any neurological disorder including Epilepsy.

Parents also completed questionnaires on pregnancy and birth complications [37] including items on birth weight ("how much did your child weigh at birth?") and smoking behaviour during pregnancy ("did you smoke during pregnancy and if so, how many cigarettes per day?"). Maternal reports of birth weight and smoking during pregnancy obtained using this measure have been shown to have excellent agreement with antenatal records (correlations of 0.98, [38]). Mothers also stated the occupation of the main earner in the household. Social class was then assigned using the UK Standard Occupational Classification (2^nd ^Edition), [39] based on the Registrar General's Social class. Social class was split into three categories; high, consisting of families with professional and managerial jobs in classes 1 and 2 (Bank managers and teachers); medium, from families with skilled occupations in classes 3 non-manual, 3 manual (e.g. nurses, insurance brokers and driving instructors) and partially from skilled workers in class 4 (e.g. Administrative workers and Post Office Workers); and low social class which consisted of families with unskilled jobs in class 5 (e.g. care workers and factory workers) and unemployed or unclassified individuals. Because of the recognised difficulties in the classification [39] of families where the main earner was in the armed forces (n = 4), we excluded these families from the analyses.

The protocol for this study was approved by the North West Multi-centre Research Committee in the UK.

### Analysis

The skewness and kurtosis of all measures was calculated. For variables where skewness and kurtosis exceeded acceptable limits, [40] data were transformed to approximate normality. Hyperactive-impulsive symptoms had to be transformed using the square root, whilst Conduct Disorder symptoms had to be transformed using the natural log plus 1. Inattentive symptoms and ODD symptoms did not require transformation. To test whether or not the indicators of environmental measures (independent variables) were associated with each of the phenotypic risk indicators (dependent variables), logistic and linear regression analyses were undertaken. Where significant associations were present, covariates which were associated with both the outcome measure and the environmental measures were included in the model. For the birth weight variables, because of differences between the national mean birth weight for singletons and those from multiple births, all individuals from multiple births (n = 9) were excluded from the analyses. All analyses were implemented using the SPSS programme (version 11; Norusis/SPSS Inc.).

## Results

The mean age of the sample was nine years and two months (SD 2 years 1 month) and 90% of subjects were male. 322 subjects (87%) met diagnostic criteria for DSM-IV ADHD (81% combined type, 6% inattentive subtype and 13% hyperactive-impulsive subtype). 239 subjects (64%) met diagnostic criteria for ICD-10 Hyperkinetic Disorder. The remaining 41 participants (11%) met diagnostic criteria for DSM-III-R ADHD. 47% of individuals met criteria for a comorbid diagnosis of ODD (n = 167), whilst 13% had a comorbid diagnosis of CD (n = 46). The mean number of DSM-IV ADHD symptoms was 14.74 (SD 2.45). For inattentive symptoms the mean number was 6.99 (SD 1.70) (range 0 to 9) while it was 7.74 (SD 1.49) (range 2 to 9) for hyperactive-impulsive symptoms. Mean numbers of ODD symptoms and CD symptoms were 3.86 (SD 2.49) (range 0 to 8) and 0.91 (SD 1.42) (range 0 to 8), respectively.

The mean birth weight (excluding individuals from multiple births) was 3286 g with a standard deviation of 621.23 g. This is similar to the UK average birth weight (for singletons) of 3300 g [41]. 163 mothers (46%) reported having smoked during pregnancy, greater number than the national UK average of between 25 and 30% [42]. Of the 345 families for whom social class could be assigned, 21% (n = 73) were of high social class, 29% (n = 101) of medium social class and the remaining 50% (n = 171) were of low social class. This group has a greater proportion of individuals of low social class in comparison to the UK general population [43].

### ADHD symptom dimensions

Analysis revealed significant associations between total number of hyperactive-impulsive symptoms with maternal smoking during pregnancy and social class, even when covariates including CD symptoms, age and verbal IQ were taken into account (see Table 1). Conversely, birth weight was not found to significantly predict number of hyperactive-impulsive symptoms (r^2 ^< 0.001, Beta = 0.05, t = 0.85, p = 0.40).

**Table 1:** Regression analysis for indicators of environmental risk predicting hyperactive-impulsive symptoms:

	**R**^**2**^	**Unstandardised**		**Standardised**		
		**Beta**	**Standard error**	**Beta**	**t**	**p-value**

Constant	-	2.28	0.42	-	5.39	<0.001
Smoking in pregnancy	0.02	0.31	0.16	0.11	1.96	0.05
Social class	0.02	0.22	0.10	0.12	2.19	0.03
Birth weight	<0.001	-0.00003	0.0001	-0.05	-0.85	0.40
CD symptoms	0.03	0.14	0.06	0.14	2.51	0.01
Age at assessment	0.01	-0.007	0.003	-0.12	-2.19	0.03
Verbal IQ	0.02	-0.003	0.002	-0.09	-1.71	0.04

To ensure that our findings were not being driven by our modest (n = 19) number of individuals with DSM-IV ADHD Inattentive subtype, we repeated the analysis with only those meeting diagnostic criteria for DSM-IV ADHD Combined type. This did not alter our findings.

Interestingly, none of the environmental measures analysed were significantly associated with total number of inattention symptoms (see Table 2).

**Table 2:** Univariate linear regression analysis for indicators of environmental risk predicting inattentive symptoms:

	**R**^**2**^	**Unstandardised**		**Standardised**		
		**Beta**	**Standard error**	**Beta**	**t**	**p-value**

Smoking in pregnancy	< 0.001	0.06	0.18	-0.02	-0.36	0.72
Social class	0.001	0.07	0.12	0.03	0.58	0.56
Birth weight	<0.001	0.00001	0.0001	-0.006	-0.11	0.91

### Comorbid Conduct Disorder symptoms and diagnosis

Social class, birth weight and maternal smoking during pregnancy significantly predicted total number of Conduct Disorder symptoms in univariate linear regression analysis. However, in multivariate analysis, including significant covariates of all the environmental indicator variables, gender, verbal IQ and total number of ADHD symptoms, the associations with social class and birth weight dropped to a trend (from 0.04 to .06 and .09, respectively), whilst the association with maternal smoking during pregnancy remained significant (see table 3). In addition, a comorbid diagnosis of Conduct Disorder was significantly predicted by maternal smoking during pregnancy (OR = 3.14, 95% CI: 1.54, 6.41, Wald = 9.95, p = 0.002) and social class (OR = 1.95 95% CI: 1.18, 3.23 Wald = 6.78, p = 0.009). These analyses controlled for each of the environmental indicator measures, age, gender, verbal IQ and total number of ADHD symptoms.

**Table 3:** Multiple regression analysis for indicators of environmental risk predicting Conduct Disorder symptoms:

	**R**^**2**^	**Unstandardised**		**Standardised**		
		**Beta**	**Standard error**	**Beta**	**t**	**p-value**

Constant	-	0.55	0.33	-	1.70	0.09
Smoking in pregnancy	0.04	0.21	0.06	0.18	3.34	0.001
Social class	0.02	0.07	0.04	0.09	1.72	0.09
Birth weight	0.007	-0.0004	0.001	-0.10	-1.89	0.06
Gender	0.02	-0.28	0.10	-0.15	-2.84	0.005
Verbal IQ	0.02	-0.004	0.003	-0.09	-1.68	0.09
ADHD symptoms	0.01	0.02	0.01	0.10	1.85	0.07

### Oppositional Defiant Disorder symptoms and diagnosis

Maternal smoking during pregnancy significantly predicted total number of ODD symptoms (see table 4). Conversely, social class and birth weight did not. When looking at a diagnosis of ODD, none of our indicators of environmental risk significantly predicted a diagnosis of ODD. Driven by clinical constructs, we decided to investigate CD and ODD separately as they are distinct (but related) disorders according to the standard classification systems DSM-IV and ICD-10. These disorders do overlap both conceptually and statistically (with r^2 ^= 0.35 between symptoms of the disorders in this sample). Therefore caution is recommended against considering the results for these disorders as wholly independent.

**Table 4:** Linear Regression analysis for indicators of environmental risk predicting Oppositional defiant symptoms:

	**R**^**2**^	**Unstandardised**		**Standardised**		
		**Beta**	**Standard error**	**Beta**	**t**	**p-value**

Smoking in pregnancy	0.01	0.58	0.26	0.12	2.20	0.03
Social class	0.004	0.19	0.17	0.06	1.14	0.26
Birth weight	<0.001	0.0003	0.0001	-0.08	-1.37	0.71

In order to ascertain which of our findings were most important, stepwise linear regression analysis was performed looking at hyperactive-impulsive and Conduct Disorder symptoms. For hyperactive-impulsive symptoms, maternal smoking during pregnancy was the most significant variable (r^2 ^= 0.02, Beta = 0.15, t = 2.52, p = 0.01). Similarly, for Conduct Disorder symptoms, maternal smoking during pregnancy was the most important factor (r^2 ^= 0.04, Beta = 0.18, t = 3.30, p = 0.001), followed by gender (r^2 ^= 0.02, Beta=-0.14, t = 2.64, p = 0.009).

## Discussion

These findings suggest that indicators of environmental risk thought to influence the diagnosis of ADHD may also have independent effects on the phenotype manifestation of ADHD within a clinical sample, something which has not been explored in previous research. Severity of hyperactive-impulsive symptoms was independently predicted by both lower social class and maternal smoking during pregnancy, with maternal smoking during pregnancy being revealed as the most important factor. Indeed, when we further analysed the data to look at quantity of cigarettes smoked, we found a dose-dependent effect, whereby the children of mothers who reported smoking more than 10 cigarettes a day during their pregnancy had more hyperactive-impulsive symptoms than those whose mothers smoked fewer than 10 cigarettes a day (r^2 ^= 0.02, Beta = 0.15, t = 2.80, p = 0.006). In contrast there was a lack of association observed between any of the indicators of environmental risk and inattentive symptom severity. This finding is in line with previous twin studies which indicate a greater influence of common environmental factors (which social class and maternal smoking during pregnancy would be) for hyperactive-impulsive symptoms, in comparison to inattentive symptoms [44]. This supports the distinction of ADHD subtypes and suggests that environmental as well as genetic factors contribute to the phenotypic heterogeneity of ADHD characterised by the type of symptoms and their severity. The results also suggest that smoking during pregnancy and environmental risk as indexed by social class may influence the aetiology of the hyperactive-impulsive dimension (or its severity) specifically, rather than all symptoms of ADHD (in a clinical sample).

Maternal smoking during pregnancy also significantly predicted total number of ODD symptoms but not a diagnosis of the disorder, possibly because such a large proportion (47%) of the sample met diagnostic criteria for ODD.

An independent, significant association was also found between maternal smoking during pregnancy and Conduct Disorder symptoms (r^2 ^= 0.04, Beta = 0.18, t = 3.34, p = 0.001), even when other significant covariates were considered. Similarly, a diagnosis of Conduct Disorder was independently associated with maternal smoking during pregnancy. Conduct disorder (but not symptoms scores) was also found to be associated with social class. These results indicate that there is an increase in the risk of a comorbid diagnosis of CD for those ADHD children whose mothers smoke during pregnancy with an odds ratio of 3.14. Furthermore, those ADHD children from a low social class family whose mothers smoke during pregnancy are at an even greater risk of being diagnosed with Conduct Disorder (more than a five-fold increase in risk). Considering the additional impairment and risks in later life for those individuals with both ADHD and Conduct Disorder, these are potentially important findings. Environmental indicators of risk which can be easily assessed, even if not causal, can help pick up children at higher risk of adverse outcomes so that risk reduction strategies and higher levels of follow up and service involvement are targeted at those most at risk.

Inevitably, this study has a number of limitations. First, we were not able to test for main effects of the environmental factors on a diagnosis of ADHD (all our subjects had a diagnosis). However, we specifically chose indicators of environmental risk which have previously been associated with ADHD and the purpose of this study was to investigate phenotypic variation within a clinical ADHD sample.

Second, it is possible that, rather than showing an association between environmental factors and comorbid CD symptoms, the results of our analysis simply revealed main effects of these factors on antisocial behaviour in general. This is certainly plausible as associations between maternal smoking during pregnancy, birth weight, social class and antisocial behaviour have previously been reported. [45,46] Unfortunately, this hypothesis can not be tested in this sample as neither an unaffected or conduct disorder alone control group were available. However, the aim of this investigation was not to find main effects of environmental measures, but rather effects within the phenotype.

Third, the failure to detect significant associations between phenotypic and indicators of environmental risk may be due to the reduced variance in ADHD symptoms in this clinical sample. However, we were interested in how the environmental factors have additional effects within the ADHD phenotype. It may even be argued that any independently significant findings are even more interesting as the power to detect them is small, although we recognise that small sample sizes can also lead to false positive findings [40].

Fourth, our indicators of environmental risk were collected retrospectively from self report. It is possible that these measures are inaccurate. Furthermore, our question assessed maternal smoking during pregnancy generally, rather than separating it into different trimesters. We therefore cannot determine whether or not there are differing effects of maternal smoking during pregnancy between those who give up at some point during the pregnancy. However, retrospective self report of maternal smoking during pregnancy has been shown to be relatively accurate, corresponding with both contemporary reports [13] and blood cotinine levels which identify tobacco products in the bloodstream. [47] Similarly, research using our measure has illustrated high levels of agreement between parental recall of birth weight and smoking during pregnancy with data from medical records. [38].

Fifth, social class is unlikely to be a proximal risk factor and is certainly only an indicator of risk. However, it is an easily obtainable indicator of risk that is readily available in clinical practice. Given these findings, there is clear justification for measuring and examining potential proximal risk factors that are indexed by social class and that may have effects on the clinical presentation of ADHD.

Sixth, as discussed previously, these indicators of environmental risk (and indeed the phenotypic measures) show covariation. It is therefore possible that our findings are the result of a separate, unmeasured factor, associated with both the environmental and phenotypic measures. In this analysis, we control for a number of possible covariates by including every environmental indicator measure in the multivariate analysis, whilst also controlling for a range of demographic and clinical variables found to be associated with the phenotypic variable.

The environmental indicator measures chosen represent easily obtainable indicators of risk, but are not necessarily causal themselves. Other designs are needed to test whether the associations are due to causal risk effects [48]. Nevertheless ascertaining such indicators of risk may be advantageous when identifying high risk subgroups of ADHD children, as well as providing a starting point for research attempting to find more proximal, causal risk factors. The majority of our sample (90%) were male and so these findings may not translate to females (although re-analysis our data with the exclusion of females did not alter our findings). Similarly, this is a clinical sample and so findings may not be generalised to individuals within the community who would meet diagnostic criteria for ADHD.

Finally, there may be a genetic link between our indicators of environmental risk and our phenotypic measures. For example, maternal smoking during pregnancy and child ADHD or conduct disorder behaviour; antisocial or ADHD mothers may be more likely to smoke during pregnancy (indeed there is extensive literature linking substance use and conduct problems e.g. [49,50]) and pass genetic risk for such behaviours to their child, regardless of their smoking behaviour. Unfortunately, we do not have data on the mothers of these children (for example psychiatric diagnoses, personality measures or IQ) and so the design of this study cannot distinguish between genetically and environmentally medicated risks. However, twin studies have indicated that maternal smoking during pregnancy has a significant environmental effect on ADHD symptoms, over and above any genetic effects [51], whilst in our previous work, we have not found any specific gene variants to be associated with both maternal smoking during pregnancy and childhood ADHD [52]. Similarly, there is some evidence to suggest that social class is associated with genes which influence Verbal IQ [53]. Although verbal IQ was included as a covariate in our analyses where relevant, the possibility that genetic factors are influencing the role of social class on hyperactive-impulsive and conduct disorder symptoms cannot be ruled out.

## Conclusion

In summary, in a clinical sample of children with ADHD, we have observed that maternal smoking during pregnancy and social class independently predict severity of hyperactive-impulsive symptoms and Conduct Disorder symptoms and diagnosis. Conversely, the indicators of environmental risk studied did not predict total number of inattentive symptoms. Previous research has focused on main effects of maternal smoking during pregnancy, birth weight and social class on diagnosis of ADHD. The findings of this study now suggest that that some of these indicators of environmental risk may also have an effect on the clinical manifestation of the disorder.

## Competing interests

AT has accepted fees from pharmaceutical companies including Janssen Cilag and Eli Lily for talks and meetings. The other authors (KL, MBMvdB & PH) declare that they have no competing interests.

## Authors' contributions

KL was responsible for the data collection, analysis and interpretation of findings. She also drafted the manuscript. PH provided statistical expertise and was involved in the interpretation of findings. He also edited the manuscript. MBMvdB was involved in the design, data collection and interpretation of the study. She also edited the manuscript. AT was responsible for the design and coordination of the study, was involved with interpretation of the findings and revised the manuscript. All authors have read and approved the final manuscript.

## Pre-publication history

The pre-publication history for this paper can be accessed here:


